# Morphological features of the acetabulum with coxa profunda in women: a retrospective observational study

**DOI:** 10.1186/s12891-024-07540-9

**Published:** 2024-05-31

**Authors:** Michitaka Kato, Takanori Ando, Shingo Mitamura

**Affiliations:** Nagoya Joint Replacement Orthopaedic Clinic, 7 Iponbashi, Takadaji, Kita-Nagoya, 481-0011 Aichi Japan

**Keywords:** Hip joint, Acetabulum, Ischium, Developmental dysplasia of the hip morphology

## Abstract

**Background:**

The morphology of coxa profunda remains inadequately understood. However, knowledge about the characteristics of the acetabulum in coxa profunda can help to predict pelvic morphology in three dimensions based on radiographic findings, as well as help to diagnose and predict hip pathologies. Therefore, this study aimed to investigate the relationship between the morphological characteristics of the pelvis and coxa profunda.

**Methods:**

We conducted a retrospective analysis including women who had undergone unilateral total hip arthroplasty. Only those with normal hip joint morphology on the opposite side, as evidenced by anteroposterior pelvic radiography showing a distance of ≥ 2 mm between the ilioischial line and acetabular floor, were included. Five parameters related to acetabular anteversion, thickness, and the position of the ilioischial line were measured using axial computed tomography at the central hip joint. The coxa profunda group (*n* = 39) and control group (*n* = 34) were compared.

**Results:**

The mean acetabular anteversion angle was 12.5° ± 4° in the control group and 22.3° ± 5.6° in the coxa profunda group. The mean thickness from the acetabular fossa to the medial wall was 7.5 ± 1.7 mm in the control group and 3.9 ± 1.2 mm in the coxa profunda group. Furthermore, the bony region representing the ilioischial line was positioned more posteriorly in the coxa profunda group than it was in the control group.

**Conclusion:**

Our findings suggest that coxa profunda in women is associated with anterior acetabular dysplasia and a thin acetabulum, in contrast to previous interpretations of excessive coverage. This insight suggests a conversion of coxa profunda from a finding of pincer-type femoroacetabular impingement to a finding of acetabular dysplasia, a revelation that also draws attention to cup positioning for total hip arthroplasty.

**Supplementary Information:**

The online version contains supplementary material available at 10.1186/s12891-024-07540-9.

## Background

Coxa profunda is a radiographic finding of pincer femoroacetabular impingement that indicates a deep acetabulum [1–5]. However, recent studies have reported that coxa profunda is a nonspecific finding, as it can also be observed in acetabular dysplasia [6–12]. The ilioischial line is located posterior to the acetabulum and corresponds to the tangent of the cortex of the posterior column [13]. When the cortex of the posterior column, which delineates the ilioischial line and the cortex of the acetabular floor, is represented two-dimensionally on radiography, the positional relationship varies among individuals. Furthermore, an ilioischial line in contact with the acetabular floor or positioned on the lateral side is classified as coxa profunda [2, 4]. Plain radiography plays a key role in identifying the morphological features of hip diseases and making the initial diagnosis. This is because it is routinely performed during the first visit or when symptoms change. Moreover, a comprehensive morphological analysis of hips with coxa profunda may help surgeons determine the appropriate diagnoses and treatments for patients. This study aimed to verify whether the abovementioned morphological pelvic characteristics are related to coxa profunda.

## Methods

### Study design

The Ethics Committee Institutional Review Board of Nagoya Orthopedic Joint Replacement Clinic waived the requirement for informed consent and approved the retrospective study (Approval no. 201,904,001). The study population included patients with asymptomatic healthy hips who visited our institution between June 2015 and February 2021. We reviewed the patients’ routine pelvic computed tomography (CT) images and supine pelvic anteroposterior radiographs acquired 5 days after unilateral hip replacement. Healthy hip joints (the hip joints contralateral to those of the hips that had undergone total hip arthroplasty [THA]) with no evidence of advanced osteoarthritis were included in this study if supine anteroposterior pelvic radiography showed a distance of ≥ 2 mm between the iliac sciatic line and acetabular floor. An orthopedic surgeon with 31 years of experience determined which patients met these inclusion criteria. In the coxa profunda group, the acetabular floor was medial to the ilioischial line. In contrast, in the control group, the acetabular floor was lateral to the ilioischial line, and Wiberg’s [14] lateral center-edge angle was ≥ 18°. The control group included patients with borderline acetabular dysplasia. Patients with acetabular dysplasia with a center-edge angle of < 18° were excluded. As the characteristics of coxa profunda may differ between men and women [12], we limited the present study to women. Overall, the exclusion criteria were as follows: male sex, prior hip surgery, advanced hip osteoarthritis grade ≥ 2 according to the Tönnis classification system [15], or severe morphological abnormalities of the femoral head. Two hips in two patients were categorized as Tönnis Grade 2 and were therefore excluded from the study. All included hips were classified as type I, according to the classification system established by Crowe et al. [16].

Following these criteria, 39 patients were included in the control group and 34 in the coxa profunda group (Table 1) to assess whether the pelvic morphology is associated with coxa profunda.

**Table 1:** Comparison of the demographic characteristics of the control and coxa profunda groups

Parameter	Control (*n* = 39 hips)	Coxa profunda (*n* = 34 hips)	*p*-value
Mean age (years)	65.4 ± 9.4	65.2 ± 7.7	0.52
Mean body mass index (kg/m^2^)	23.9 ± 3.4	22.6 ± 2.8	0.35

^a^ Values are presented as mean ± standard deviation.

**Fig. 1:**
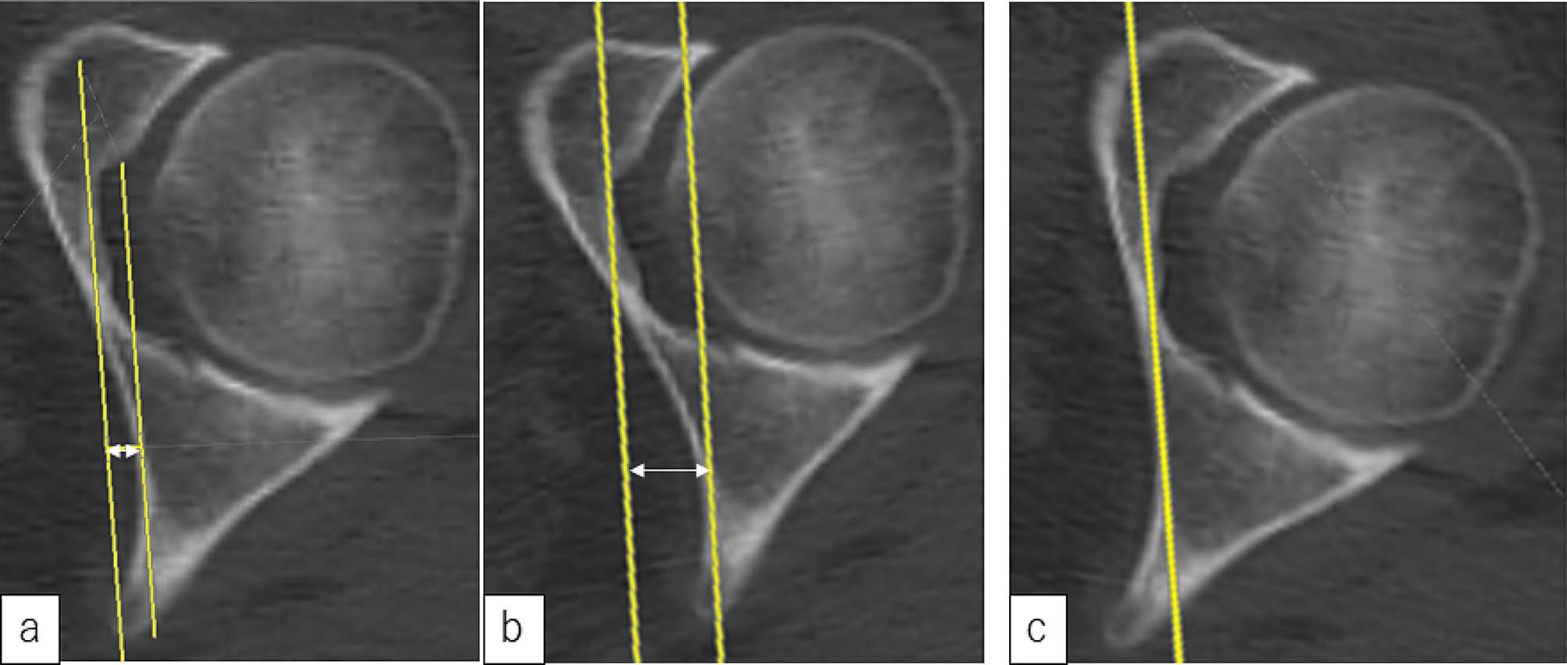
Axial CT image at the hip center level in a case of coxa profunda. **a** Original image. A line with a 4° incline was created to mimic the X-ray irradiation line, which is tangential to the acetabular floor and acetabular medial wall. The distance between these two parallel lines approximates the distance between the ilioischial line and acetabular floor on the anteroposterior pelvic radiograph. **b** The two same parallel lines were created on the original image with 10° internal rotation. The distance between the ilioischial line and the acetabular floor is increased. **c** The original image was rotated 10° externally. The cortical bone in contact with the line in image **b** is located more posterior to the ischium than in image **c**. CT, computed tomography

To explore the characteristics of an acetabulum with coxa profunda, we identified three pelvic morphological features that we predicted would increase the likelihood of the ilioischial line being located laterally on the acetabular floor. The first feature was the cortical bone of the posterior column, which represents the ilioischial line, being located more posteriorly in the pelvis. However, the anteversion of the acetabulum should be noted; thus, if the cortical bone of the posterior column is located more posteriorly, it is more probable that the ilioischial line will be located laterally. The second morphological feature was the anteversion of the acetabulum. The acetabular floor is usually located anterior to the cortical bone of the ischium, which represents the ilioischial line. Thus, if the acetabulum is notably anteverted, the posterior column that delineates the ilioischial line is more likely to be lateralized against the acetabular floor, depending on the relative position of the acetabulum. When the CT axial image at the hip center level was internally rotated, considerable anteversion of the acetabulum was observed, and the cortical bone area indicating the ilioischial line was positioned more posteriorly and laterally to the acetabular floor. Conversely, the opposite was observed when external rotation was applied. Our first and second predictions were formulated based on our observation of these phenomena (Fig. 1). The final feature was the thinness of the bone extending from the acetabular floor to the medial wall, with a thinner bone at the acetabular fossa suggesting a more medial position of the acetabular floor.

### Imaging

Plain pelvic radiographs and axial CT images were routinely obtained on postoperative day 5. The radiographs were obtained with the patient in the supine position. The X-ray tube (Radnext32; FUJIFILM, Tokyo, Japan) was positioned parallel to the floor and centered on the pubic symphysis, with a focal film length of 100 cm. The average distance from the hip joint to the film was 10.7 cm, corresponding to approximately 110% magnification on the radiographs [17].

Routine CT examinations evaluated occult fractures and the cup and stem anteversion position. A 16-row multi-CT model (ECLOS; Hitachi, Tokyo, Japan) was used for all examinations. Multiplanar reformation axial images at 3-mm intervals from the volumetric CT were obtained parallel to the inter-teardrop line. The slice with the largest anteroposterior metal femoral head diameter was selected to determine the location of the ilioischial line. All measurements were made using a digital viewer.

### X-ray beam angle

The perspective projection angle of the X-ray beam around the ilioischial line was calculated to be approximately 3.8° in a previous study [17]; therefore, we set the projection angle at 4° to the perpendicular line for simplification. The region of the bone that overlaps the line (line α) tangential to the medial wall of the acetabulum with a perspective projection angle of 4° tilted to the perpendicular line to the table on the axial CT image represents the ilioischial line on radiography [17].

### Radiographic evaluation

**Fig. 2:**
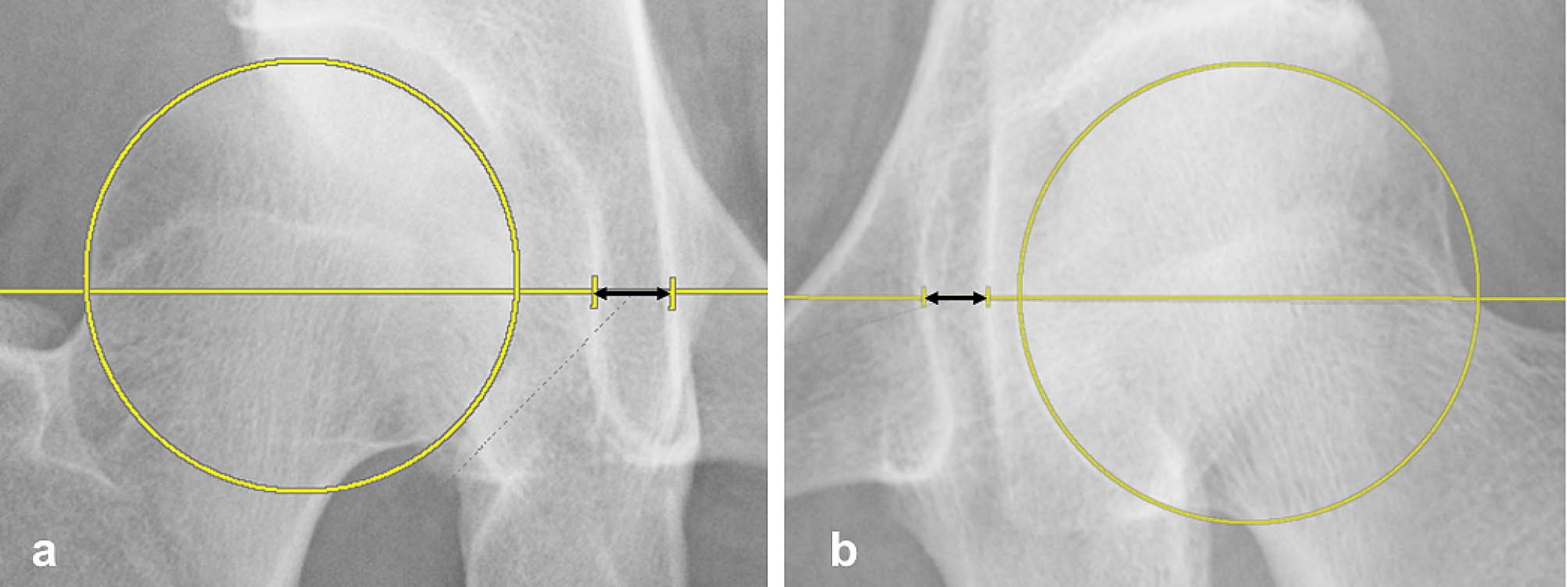
Measurement of the distance between the ilioischial line and acetabular floor. The distance between the ilioischial line and acetabular floor (black double arrow) was measured at the hip center, parallel to the inter-teardrop line. **a** A coxa profunda-negative case (66-year-old woman): the measured distance from the ilioischial line and epiphysis to the acetabular floor is represented by a positive number. **b** A coxa profunda-positive case (56-year-old woman): the measured distance from the ilioischial line and epiphysis to the acetabular floor is represented by a negative number

Coxa profunda was defined as an acetabular floor that touches or is medial to the ilioischial line on pelvic radiography [2, 4]. The lateral center-edge angle was measured as a radiological indicator of acetabular dysplasia or over-coverage. The distance between the ilioischial line and the acetabular floor parallel to the inter-teardrop line at the hip center was measured (Fig. 2).

### CT imaging measurements

**Fig. 3:**
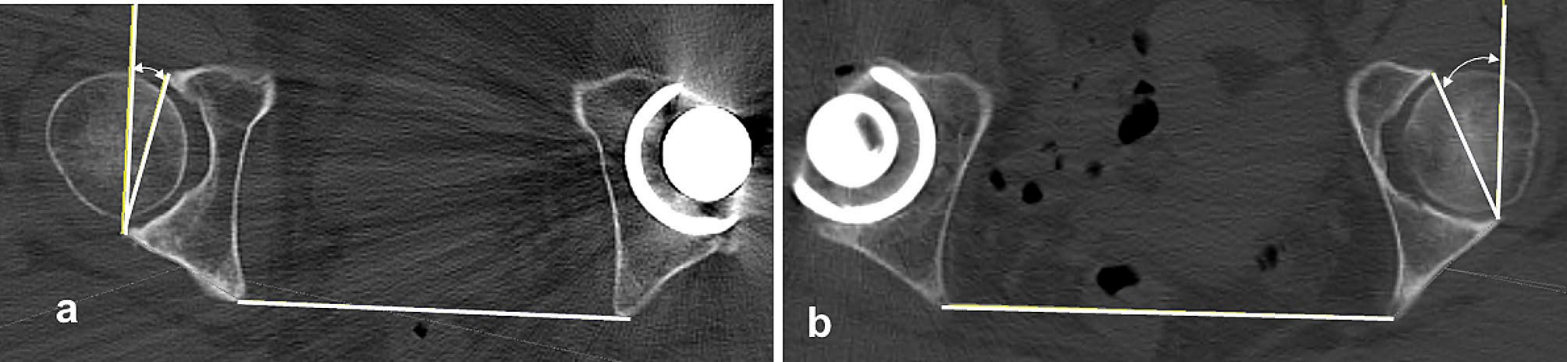
Measurement of the acetabular anteversion angle. The CT axial image at the hip center was selected. The acetabular anteversion angle (white curved double arrow) was defined as the angle between a reference line perpendicular to a line connecting the posterior ischia and a line connecting the posterior and anterior margins of the acetabulum. **a** A coxa profunda-negative case. **b** A coxa profunda-positive case. CT, computed tomography

Axial CT slices with the largest femoral head diameters were selected to measure each parameter at the hip center level. The acetabular anteversion angle was measured as the angle between a reference line perpendicular to a line connecting the posterior ischia and a line connecting the posterior and anterior acetabular margins (Fig. 3).

**Fig. 4:**
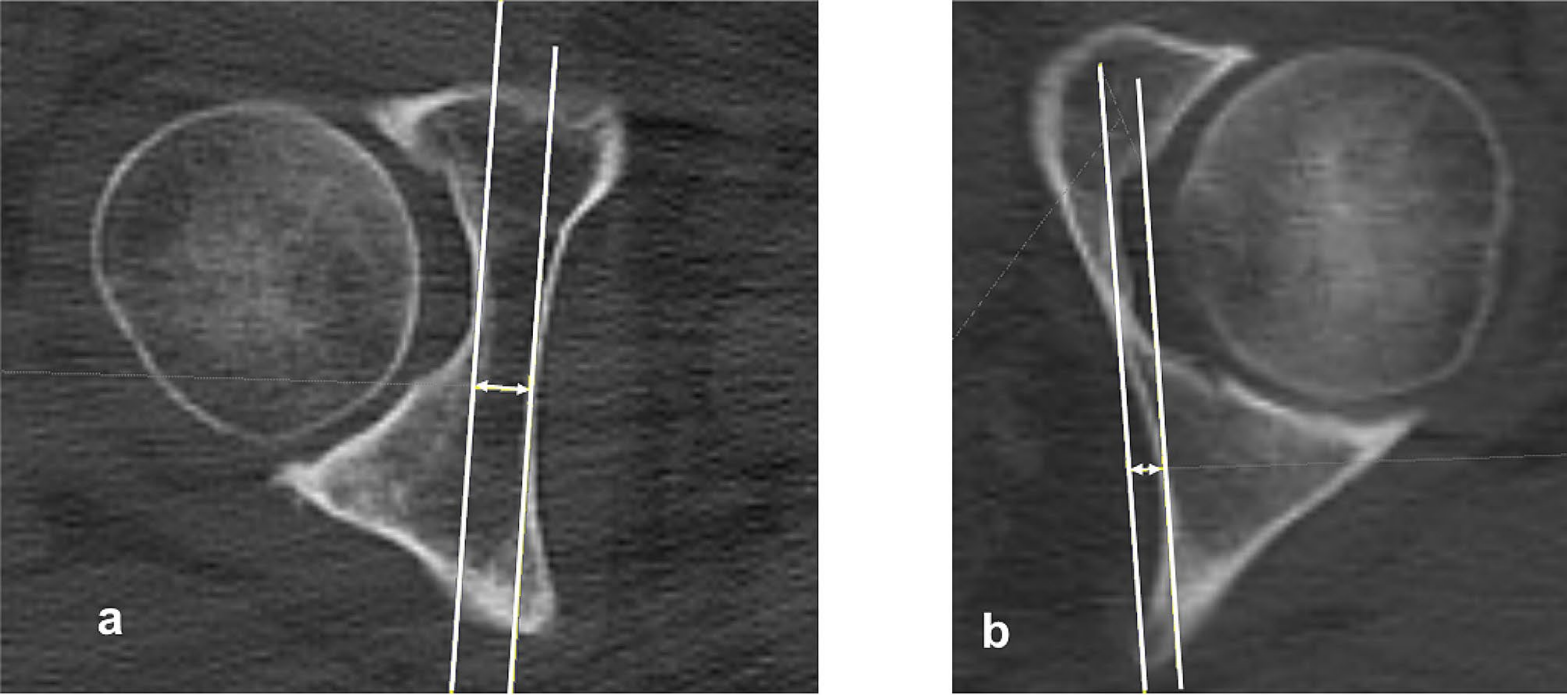
Measurement of the distance between line α and a line parallel to it and tangential to the acetabular floor. The bony region on axial CT images, which overlaps with the line (line α) with a perspective projection angle (inclined 4° to the vertical line) to the ilioischial line during radiography and tangential to the medial acetabular wall, represents the ilioischial line on the radiograph. The distance between line α and a line parallel to line α and tangential to the acetabular floor (white double arrow) was measured. **a** A coxa profunda-negative case. **b** A coxa profunda-positive case. CT, computed tomography

We measured the distance between line α and a line parallel to it and tangential to the acetabular floor to verify that it was approximately the same as the distance between the acetabular floor and the ilioischial line on the anteroposterior pelvic radiograph (Fig. 4).

**Fig. 5:**
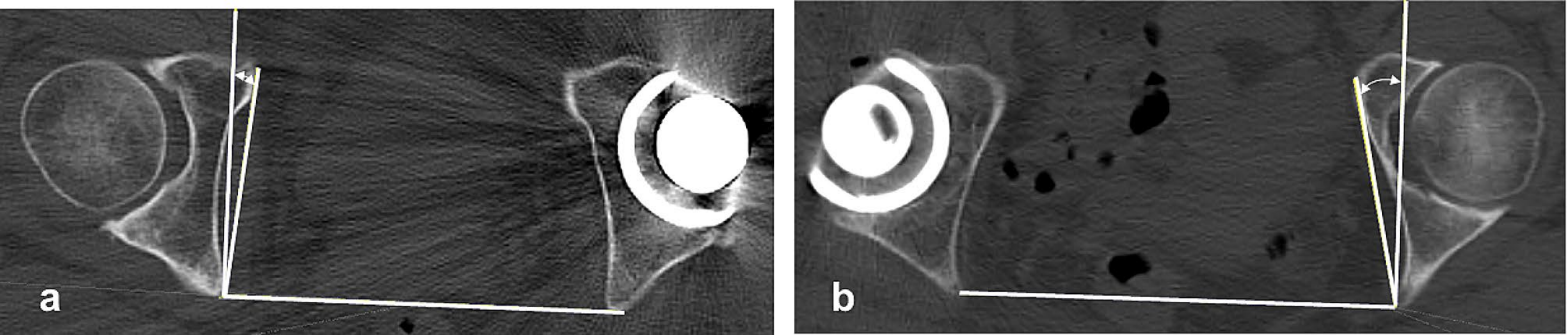
Measurement of the medial wall anteversion angle. The medial wall anteversion angle (white curved double arrow) at the hip center was measured as the angle between a reference line perpendicular to a line connecting the anteromedial margin of the pubis and the posteromedial margin of the ischium. **a** A coxa profunda-negative case. **b** A coxa profunda-positive case

The medial wall anteversion angle at the hip center was measured as the angle between a reference line perpendicular to the line connecting the anteromedial margin of the pubis and the posteromedial margin of the ischium (Fig. 5).

**Fig. 6:**
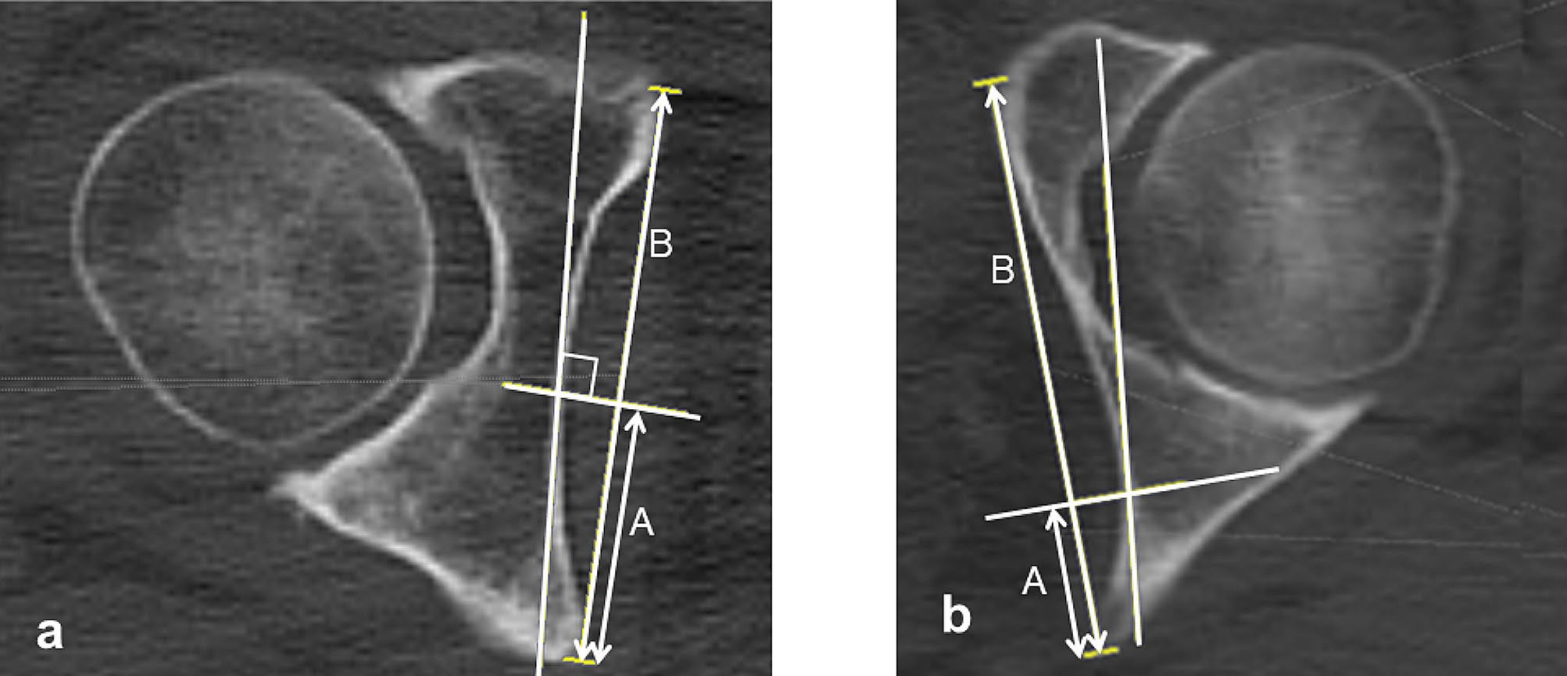
Measurement of the posterior ratio of the ilioischial line point on the axial CT slice The posterior ratio of the ilioischial line point on the axial CT slice was obtained by dividing the length from the center point of the cortical bone, indicating the ilioischial line to the posteromedial margin of the ischium (**A**), by the length from the anteromedial pubis margin to the posteromedial ischial margin (**B**). **a** A coxa profunda-negative case. **b** A coxa profunda-positive case. CT, computed tomography

The posterior ratio of the ilioischial line in the axial CT slice was obtained by dividing the length from the center of the cortical bone, which indicates the ilioischial line, to the posteromedial ischial margin (A), by the length from the anteromedial pubis margin to the posteromedial ischial margin (B) (Fig. 6).

**Fig. 7:**
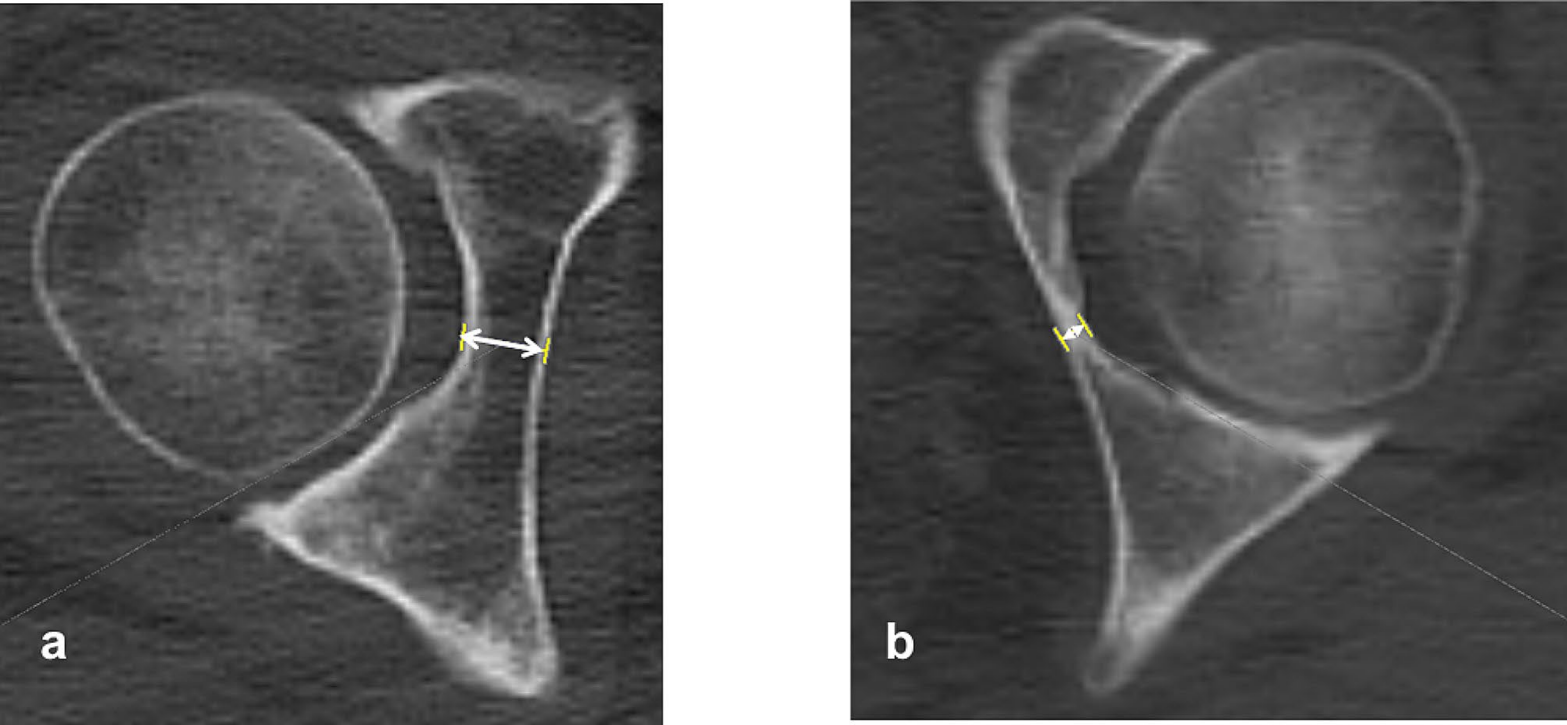
Measurement of the thickness of the medial acetabular wall. The thickness of the medial acetabular wall was measured as the distance from the acetabular floor to the medial cortex at the hip center. **a** A coxa profunda-negative case. **b** A coxa profunda-positive case

The bone thickness at the acetabular floor was measured as the distance from the most medial point of the acetabular floor to the medial wall (Fig. 7).

**Fig. 8:**
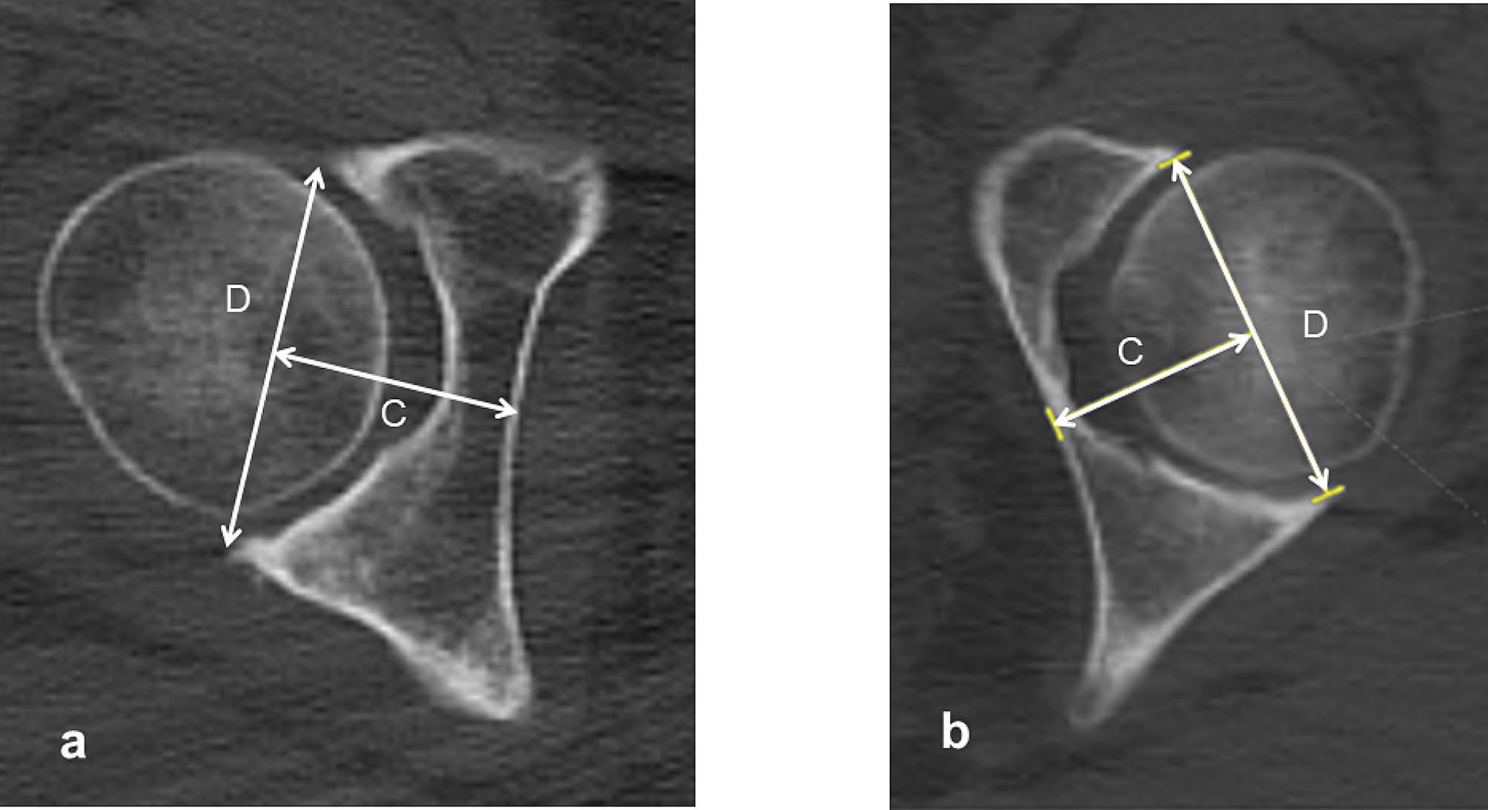
Measurement of the acetabular depth to medial wall ratio. The acetabular depth ratio denotes the ratio of acetabular depth to the medial wall (**C**) to the acetabular width (**D**) in the axial plane passing through the femoral head center. **a** A coxa profunda-negative case. **b** A coxa profunda-positive case

The axial acetabular depth ratio to the medial wall was determined as the ratio of the acetabulum to medial wall depth (C) to the acetabular width (D) (Fig. 8).

### Statistical analysis

A clinically experienced radiologist obtained the measurements twice, at least 2 weeks apart, in a blinded fashion. The averages of each pair of measurements were used. Furthermore, the intra-observer reliability of the CT measurements was assessed using intraclass correlation coefficients, which was good (range: 0.94–0.99). Three independent observers conducted two sessions at least 2 weeks apart, and the interobserver reliability on 10 hips was assessed. Kappa values of 0.82 for intra-observer reliability and 0.82 for interobserver reliability suggested an almost perfect agreement.

Unpaired *t*-tests were performed to compare continuous parameters between both groups (Shapiro–Wilk W test, F test). The significance level was set at < 0.05 for all tests. The Bland–Altman method [18] was used to analyze the agreement between the radiographs and CT in terms of the distance between the ilioischial line and the acetabular floor. The Statistical Package for Social Sciences® (version 26.0; IBM Corp., Armonk, NY, USA) was used for all statistical analyses and to create the Bland–Altman plots.

Since there is no precedent study analyzing the morphology of coxa profunda, the sample size was validated in a post hoc analysis using G*power (version 3.1.9.7) [19, 20]. The effect size of each survey item was examined based on the mean and standard deviation values of each group. The center-edge angle was the smallest, at 0.99, and the power was 0.99, with the alpha error probability set at 0.05 in the post hoc analysis; this indicated a sufficient sample size.

## Results

There were no significant differences in demographics between the two groups (Table 1). The radiography and CT parameters are presented in Table 2**(see Additional file 1)**. Significant differences were observed between the two groups in all parameters except the center-edge angle. The coxa profunda group had a significantly larger acetabular anteversion angle than the control group, with a mean difference of 9.8°. The bone extending from the acetabular floor to the medial wall was, on average, 3.6 mm thinner in the coxa profunda group than it was in the control group. The region of bone representing the ilioischial line was located 12% more posterior to the anteroposterior length of the medial wall in the coxa profunda group than it was in the control group.

**Table 2:** Comparison of CT measurements between the control and coxa profunda groups

Parameters		Control (*n* = 38 hips)	Coxa profunda (*n* = 34 hips)		*p*-value
Radiography	CE angle	28.4 ± 5.7		29.4 ± 6.4	0.28
	Acetabular floor to ilioischial line distance (mm)	5.2 ± 1.7		-3.9 ± 1.5	< 0.001
	0.91 × acetabular floor to ilioischial line distance (mm)	4.8 ± 1.6		-3.5 ± 1.4	< 0.001
Axial CT image	1: Acetabular floor to ischial wall distance (mm)	4.1 ± 1.6		-2.8 ± 1.4	< 0.001
	2: Acetabular anteversion angle	12.5 ± 4°		22.3 ± 5.6°	< 0.001
	3: Pelvic anteversion angle	3.9 ± 5°		9.3 ± 4.6°	< 0.001
	4: Ilioischial line cortex point to anteroposterior ratio	0.39 ± 0.12		0.27 ± 0.07	< 0.001
	5: Thickness of the acetabular fossa to the medial wall (mm)	7.5 ± 1.7		3.9 ± 1.2	< 0.001
	6: Axial acetabular depth to medial wall ratio	0.61 ± 0.06		0.56 ± 0.05	< 0.001

^a^ Values are presented as mean ± standard deviation. CE, center-edge; CT, computed tomography.

The CT images of typical coxa profunda and coxa profunda-negative cases are presented in Figs. 2, 3, 4, 5, 6, 7 and 8. In patients with coxa profunda, the acetabulum showed greater anteversion and the acetabular bone was thinner.

**Fig. 9:**
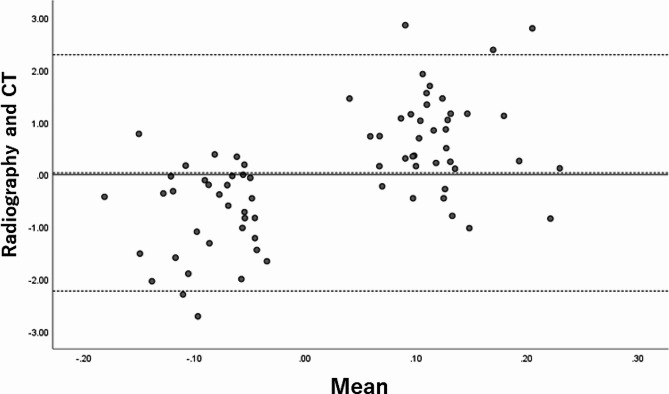
Bland–Altman plot. A Bland–Altman plot of the acetabular floor to the ischial wall and 0.91 × acetabular floor to ilioischial line distances

The mean difference between the acetabular floor to ischial wall distance and 0.91 × acetabular floor to ilioischial line distance, corrected for radiographic magnification, was 0.87 ± 0.72 mm. A Bland–Altman plot of the acetabular floor to ischial wall distance and 0.91 × acetabular floor to ilioischial line distance revealed five outliers (Fig. 9).

## Discussion

When a two-dimensional bony structure revealing the acetabular floor and ilioischial line is projected on a frontal pelvic radiograph, coxa profunda is considered to be present if the acetabular floor is tangential or medial to the ilioischial line [2, 4].

The novel aspects of this study were its identification of cortical bone showing ilioischial lines on CT, as well as the determination and verification of the morphological conditions of acetabula showing coxa profunda. As a result, we were able to prove that the morphological features of acetabula showing coxa profunda are associated with anterior acetabular dysplasia and acetabular thinness. Regarding the presentation of the ilioischial line on the pelvis when viewed in three dimensions, previous studies have suggested that the ilioischial line is located on the medial acetabular wall of the ischium [13, 17]. We used the perspective projection angle (4°) of the X-ray beam around the ilioischial line, which was calculated using a trigonometric function in our previous study [17], for the line tangential to the ischial cortex that reveals the ilioischial line on CT images. The mean difference between the distances from the ilioischial line to the acetabular floor on radiographs and distances from the acetabular floor to the ischial wall on CT was 0.87 mm, indicating good approximation. Our results are supported by our previous findings [17], which showed that the region of the bone on the axial CT image that overlaps the line (line α) tangential to the medial wall of the acetabulum, with a perspective projection angle of 4° tilted to the perpendicular line to the table, represents the ilioischial line on radiography [17].

To the best of our knowledge, this is the first study on coxa profunda based on the relationships between the three specified morphological features of the acetabulum and coxa profunda.

Although coxa profunda is considered an indicator of radiographic acetabular over-coverage or pincer femoroacetabular impingement, previous studies have suggested that coxa profunda is a nonspecific finding in asymptomatic hips and hips with various disorders, such as hip dysplasia [7, 8, 10, 11]. Boer et al. noted that coxa profunda is associated with a high rate of arthroplasty and may be responsible for osteoarthritis [21]. Fujii et al. [10] reported that acetabular anteversion is related to the morphology of the coxa profunda, similar to the results of this study. Extensive acetabular anteversion is expected to result in lesser anterior coverage, suggesting that it is related to dysplasia, which is characterized by minimal anterior wall coverage. This may explain why arthroplasty is more likely to be performed on hip joints with coxa profunda than on normal hip joints [10]. The morphology of the acetabular bone in patients with coxa profunda is characterized by strong anteversion, a thin acetabular bone, and a higher arthroplasty rate. The distances between the ilioischial line and acetabular floor of coxa profunda in participants included in this study were all ≥ 2 mm, strongly reflecting the characteristics of coxa profunda. The average acetabular anteversion angle was 22.3°; the angle was ≥ 25° in 26.5% and ≥ 30° in 8.8% of all included cases. The appropriate cup anteversion angle for THA should be 10–30°. When the cups are placed in innate acetabular anteversion, the cup anteversion may deviate from the safe zone, resulting in postoperative THA complications, such as dislocation or implant impingement. In addition, if the target placement angle is 20°, the cup may protrude beyond the anterior acetabular wall, causing iliopsoas impingement. The thinness of the acetabular bone makes adjusting the axial protrusion of the cup by reaming it slightly deeper challenging. Ueno et al. reported that an axial cup protrusion of 12 mm was an independent predictor of symptomatic iliopsoas impingement, and that higher acetabular anteversion was associated with cup protrusion [22].

When performing THA on hips with coxa profunda, the surgeon should take care in cases where there is a thin acetabular medial wall. Miettinen et al. [23] reported that early aseptic loosening of the cementless cup was significantly more frequent in Dorr type A acetabula [24], which are considered to have a thin medial wall, than in Dorr type B acetabula [24], which have a thicker medial wall; this suggests that acetabula with coxa profunda may also have an increased risk of early aseptic loosening of the cementless cup. Future research should focus on the relationship between Dorr type A acetabula and coxa profunda.

There are some limitations to this study. First, the study focused on healthy hip joints of patients who underwent unilateral THA. We confirmed that the patients had no history of hip disease, osteoarthritis, or other hip abnormalities and determined that the acetabula in this study would be appropriate for morphological evaluation. However, there were many bilateral hip osteoarthritis cases, and the hips on the opposite side to the THA joint may have had pre-osteoarthritis. In addition, the patients’ average age increased, and age-related changes in lumbar spine alignment may have affected the pelvic tilt. These changes are considered to alter the pelvic morphology. Second, the frontal pelvic radiography and CT were not based on the anterior pelvic plane, and it is difficult to perform anteroposterior pelvic radiography with an accurate anterior pelvic plane. Consequently, the supine positions of the pelvis during radiography and CT may have differed, and deviations within a few degrees may have occurred. The Bland–Altman plot revealed five outliers, which we speculate resulted from mismatches in pelvic rotation. Third, the cohort of this study was limited to Japanese women. Previous studies have revealed differences in the morphological characteristics of the hip joints of Asians and Caucasians [25, 26]; for example, Asians have shallower acetabula than Caucasians. Nevertheless, we believe that our observations in this study are based on logical hypotheses, are not affected by race, and can be generalized to both Caucasian and Asian patients. However, men and women have different pelvic morphologies, and coxa profunda is more common among women than among men [8, 11]. The sex ratios in the coxa profunda and coxa profunda-negative groups differed from those in the general population since we limited our analysis to female patients. Therefore, further investigation is needed to determine whether similar results can be obtained in studies including different races and sexes.

## Conclusion

The results of this study suggest that coxa profunda observed by hip radiography without significant abnormal findings is related to a type of dysplasia in which the acetabulum is anteverted and has less anterior coverage than normal. The presence of coxa profunda also suggests a thin acetabulum and lack of anterior acetabular coverage; this should prompt careful attention to early loosening during the placement of cementless cups in THA, and measures to prevent psoas impingement should be considered.

### Electronic supplementary material

Below is the link to the electronic supplementary material.

Supplementary Material 1

## Data Availability

The data that support the findings of this study are available from the corresponding author, MK, upon reasonable request.

## Abbreviations

CT: computed tomography
THA: total hip arthroplasty
